# Design, Synthesis and Evaluation of the Antibacterial Enhancement Activities of Amino Dihydroartemisinin Derivatives

**DOI:** 10.3390/molecules18066866

**Published:** 2013-06-10

**Authors:** Chong Wu, Jian Liu, Xichun Pan, Wenying Xian, Bin Li, Wei Peng, Jingfang Wang, Dacheng Yang, Hong Zhou

**Affiliations:** 1 Department of Pharmacology, College of Pharmacy, Third Military Medical University, Chongqing 400038, China; 2 School of Chemistry and Chemical Engineering, Southwest University, Chongqing 400715, China; 3 Shanghai Center for Systems Biomedicine, Shanghai Jiao Tong University, Shanghai, 200240, China

**Keywords:** dihydroartemisinin, derivatives, antibiotic resistance, antibacterial activity, synergistic effect, β-lactam antibiotic

## Abstract

Artemisinin (ART) and its derivatives artesunate (AS), dihydroartemisinin (DHA) are a group of drugs containing a sesquiterpene lactone used to treat malaria. Previously, AS was shown to not have antibacterial activity but to significantly increase the antibacterial activities of β-lactam antibiotics against *E. coli.* Herein, molecular docking experiments showed that ART, AS and DHA could dock into AcrB very well, especially DHA and AS; both DHA and AS had the same docking pose. The affinity between AS and AcrB seemed weaker than that of DHA, while the succinate tail of AS, which was like a “bug”, could extend in the binding pocket very well. Imitating the parent nucleus of DHA and the succinate tail of AS, twenty-one DHA derivatives **4a**–**u** were designed and synthesized. Among them, seventeen were new compounds. The synergistic effects against *E. coli* AG100A/pET28a-AcrB showed among the new structures **4k**, **4l**, **4m**, **4n**, and **4r** exhibited significant synergism with β-lactam antibiotics although they had no direct antibacterial activities themelves. The bacterial growth assay showed that only 4k in combination with ampicillin or cefuroxime could totally inhibit bacterial growth from 0 to 12 h, demonstrating that **4k** had the best antibacterial enhancement effect. In conclusion, our results provided a new idea and several candidate compounds for antibacterial activity enhancers against multidrug resistant *E. coli*.

## 1. Introduction

Drug resistance of pathogenic bacteria to antimicrobial agents is a serious threat to public health. In Northeast China, more than half of the main pathogenic bacterial strains isolated from patients in intensive care units are resistant [1]. In Harbin (China), 52% of clinical isolates were Gram-negative bacteria [2]. In Shanghai, 61% of clinical isolates were Gram-negative bacteria [3], the main strains included *Escherichia coli* (*E.* *coli*), *Pseudomonas aeruginosa* (*P*. *aeruginosa*) and *Acinetobacter baumannii*, and most of them were multidrug-resistant (MDR).

Resistance Nodulation cell Division (RND) superfamily-derived efflux pumps (such as AcrB in *E. coli* and MexB in *P*. *aeruginosa*) play a very important role in producing multi-drug resistance in Gram-negative bacteria [4,5]. AcrAB-TolC, a three-component efflux pump system, is the most prevalent efflux pump of the RND superfamily [6]. AcrB, the efflux transporter of the three-component efflux pump, is an ATP-dependent homotrimer which locates in the inner membrane [7,8]. It can capture different kinds of substrates in the periplasmic space and transports them into the outer membrane channel, TolC [9]. AcrA is an adapter connects AcrB with TolC [10].

The substrates of AcrAB-TolC include noxious substances like dyes, detergents, bile salts and small organic molecules, as well as different kinds of antibiotics (such as β-lactam antibiotics, fluoroquinolones, macrolides, tetracyclines and chloromycetin). Overexpression of AcrAB-TolC confers resistance to antibiotics [11]. Therefore, AcrA, AcrB and TolC, but especially AcrB, are now presumed to be good targets for developing efflux pump inhibitors (EPIs). Unfortunately, until now there is no drug in the clinic targeting the three-component efflux pump.

Artemisinin (ART), a natural sesquiterpene lactone isolated from *Artemisia annua* L., is used as antimalarial agent. Its derivatives include dihydroartemisinin (DHA), artesunate (AS), arteether, *etc*. Previously, we demonstrated that neither ART, AS or DHA had any antibacterial ability, but AS and DHA significantly increased the *in vitro* and *in vivo* antibacterial activities of β-lactam antibiotics against *E. coli*. The drug target was thought to be AcrB because AS lost its enhancement activities in AcrB-knocked out *E. coli*. Therefore, AS was thought to be an EPI [12]. Because all of derivatives possessed the same parent nucleus structure and differed only in their side chains, these side chains were presumed to determine the antibacterial enhancement. 

Based on the above consideration, we thought more active EPIs could be obtained if the side chain was further modified. In the present experiments, molecular docking experiments were first carried out, and then a series of DHA amino derivatives were synthesized and their bioactivities were evaluated in order to get more active EPIs.

## 2. Results and Discussion

### 2.1. Computer-Predicted Ligand Binding to AcrB

**Figure 1:**
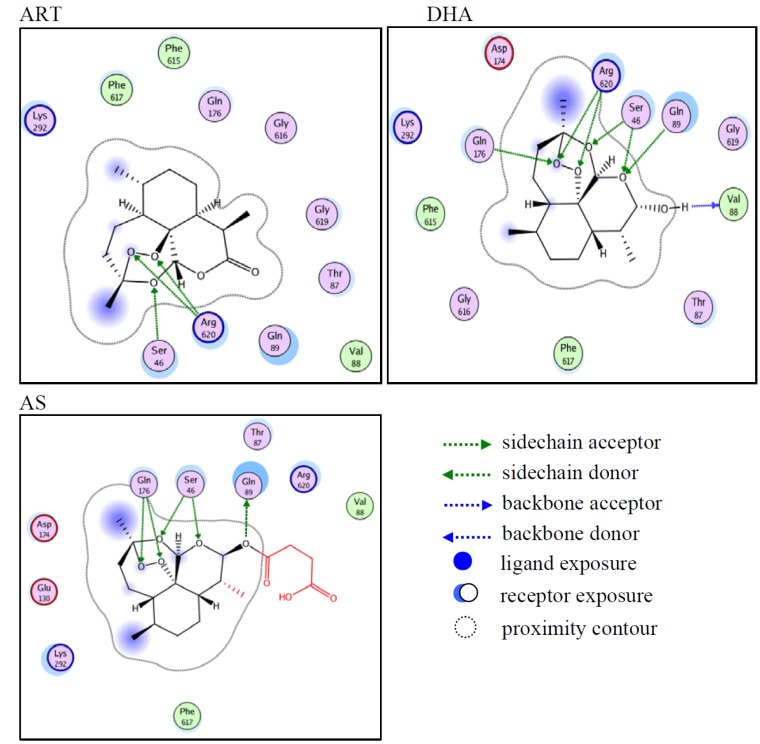
Proposed molecular docking details between AcrB and the ligands. The interactions of AcrB:ART, AcrB:DHA, and AcrB:DHA are respectively shown in A, B, and C. The residues closed to proximity contour of ligands are shown in colored circles. The ligands are shown in plane carbon skeleton models with the proximity contours. The proposed hydrogen bonds between residues of AcrB and ligands are shown in arrows. Both ligand and receptor exposures are shown in circular shadows.

To investigate whether and how ART, DHA and AS bound AcrB, molecular docking experiments were carried out based on a previous report [13,14]. Herein, the reported AcrB crystal structure was used as the rigid “receptor”, and the binding to the receptor of three candidate ligands which were set as flexible conformations was examined by the MOE program. Because the three candidate ligands had same parent sesquiterpene lactone structure, the three were put into the same domain of AcrB to trigger the docking processes. The London dG scores of ART, DHA and AS were −13.28, −14.76 and −15.02, respectively. The above docking results suggested that the three candidate ligands could dock into AcrB very well, especially DHA and AS. In the most favored docking mode, the three ligands were revealed to be all bound to the upper portion (farther away from the membrane surface) of the drug-transport pocket (Figure 1). The shape of upper portion was like a diminishing “cave”, forming a small drug-transport tunnel. Therefore, ART, DHA and AS could be defined as cave-binders and supposed to block the drug efflux.

Although the three ligands had the same parent sesquiterpene lactone structure, only DHA and AS had same docking poses, while ART was different from its two derivatives (Figure 1). The parent sesquiterpene lactone structure of DHA could tightly bind to Ser46, Val88, Gln89, Gln176 and Arg620, which were around the entrance surface to the “cave”. DHA formed seven hydrogen bonds via the four heterocyclic oxygen atoms of the parent structure with the residues mentioned above, so the ligands seemed to cap the “cave” entrance. The parent structure of AS similarly bound to the entrance surface of the “cave”, forming five hydrogen bonds with Ser46, Gln89 and Gln176.

AcrB contains three subdomains, including a TolC-docking subdomain (located in the N-terminal end) and a porter (middle) of the periplasmic domain, as well as a transmembrane domain (located in the C-terminal end) which were also identified before [14]. A drug-transport pocket involved in the porter had been defined as the main substrate efflux tunnel [9,15]. The binding between AS and AcrB seemed weaker than that of DHA, while the succinate tail of AS, like a “bug”, could extend in the binding pocket very well. This might help AS block the drug efflux and confer antibacterial enhancement effects. The parent structure of ART could only form three hydrogen bonds with Ser46 and Arg620, and ART had no “bug” like AS, so the binding between AS and AcrB was weaker than between DHA and AS. The above docking results suggested the structures of DHA and AS were good references to obtain more effective EPIs.

### 2.2. Synthesis of DHA Derivatives

**Scheme 1:**
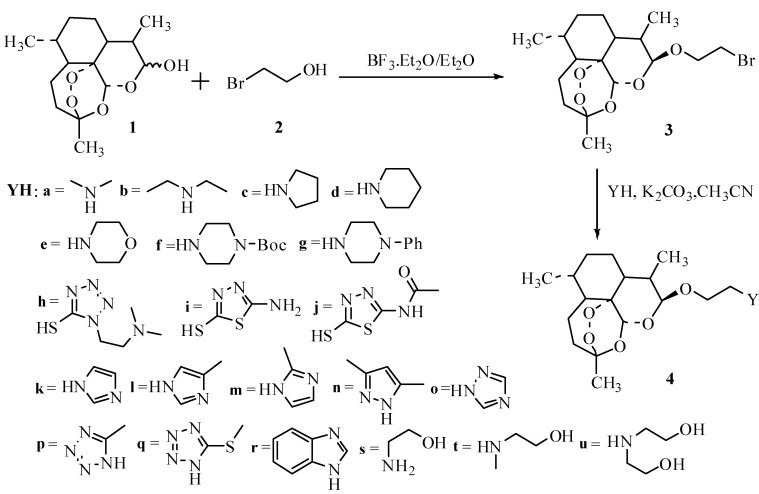
Synthetic route to DHA amino derivatives.

**Figure 2:**
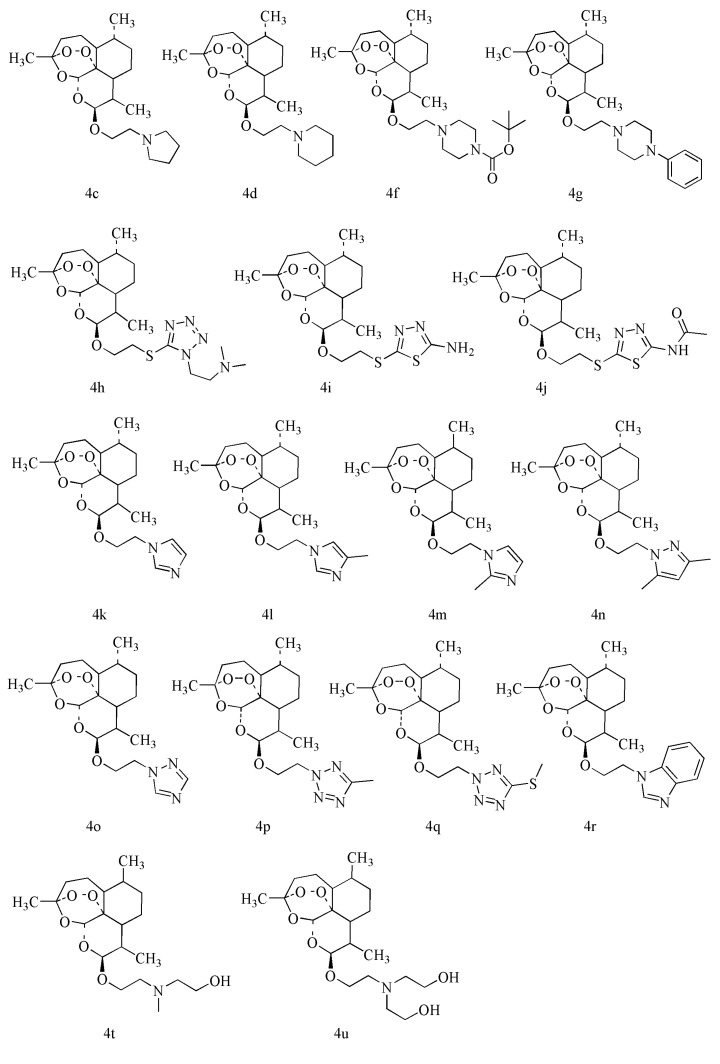
The structures of 17 new compounds.

Based on above results and suggestions, twenty-one DHA derivatives **4a**–**u** were designed in order to imitate the parent nucleus of DHA and the succinate tail of AS, and then the derivatives were synthesized. The synthetic route to the DHA derivatives is illustrated in Scheme 1. Among these derivatives, seventeen were new (Figure 2).

### 2.3. Antibacterial Enhancement Activity of DHA Derivatives

#### 2.3.1. DHA Derivatives Have no Directly Antibacterial Activity

The results showed *E. coli* AG100A/pET28a-AcrB was resistant to ampicillin and cefuroxime, with MICs of 2 and 512 μg/mL, respectively. The MICs of most of the DHA derivatives were more than 1,024 μg/mL, and some were more than 2,048 μg/mL (Table 1), which was thought to be no clinically significance since the MIC was so high, and suggesting these DHA derivatives had no direct antibacterial activity.

**Table 1:** MIC values of DHA derivatives, ampicillin andcefuroxime against *E. coli* AG100A/pET28a-AcrB. The MIC values were taken as the lowest drug concentrations at which observable growth was inhibited. Since **4f**, **4g**, **4j**, and **4q** were insoluble in LB broth, there was no MIC value for them. AMP, ampicillin; CFX, cefuroxime; **4a**–**u**, DHA derivatives.

Agents	MIC (μg/mL)		Agents	MIC (μg/mL)
AS	>1024		**4m**	512
**4a**	2048		**4n**	>2048
**4b**	>2048		**4o**	1024
**4c**	>2048		**4p**	>2048
**4d**	>2048		**4r**	1024
**4e**	1024		**4s**	1024
**4h**	>2048		**4t**	2048
**4i**	2048		**4u**	1024
**4k**	512		AMP	32
**4l**	512		CFX	512

#### 2.3.2. DHA Derivatives Have Antibacterial Enhancement Activities

The results from the drug susceptibility assay showed that the fractional inhibitory concentration index (FICI) values produced by five DHA derivatives (compounds **4k**, **4l**, **4m**, **4n**, and **4r**) in combination with ampicillin and cefuroxime were less than or equivalent to 0.5 (Table 2), demonstrating that these five DHA derivatives could increase the antibacterial activities of ampicillin and cefuroxime. Importantly, these five DHA derivatives in combination with ampicillin and cefuroxim produced a lower FICI, suggesting these derivatives possibly had stronger antibacterial enhancement activities. Among these five DHA derivatives, **4k**, **4l**, and **4m** possessed more significant enhancement for both ampicillin and cefuroxime; FICIs were lower than 0.2 (Table 2). Therefore, these three derivatives were further investigated in subsequent experiments to observe their influence on bacterial dynamic growth.

**Table 2:** FICI values for DHA derivatives in combinations with ampicillin andcefuroxime against *E. coli* AG100A/pET28a-AcrB. Synergistic effects of different concentrations of DHA derivatives in combination with ampicillin and cefuroxime were evaluated using chequerboard method. FICI values were interpreted as follows: <0.5 = synergy; 0.5–4.0 = no interaction; and >4.0 = antagonism. AMP, ampicillin; CFX, cefuroxime.

Drug concentrations	FICI
1/4 MIC AS + 1/4 MIC AMP	0.50
1/4 MIC AS + 1/16 MIC CFX	0.31
1/32 MIC **4k** + 1/16 MIC AMP	0.09
1/64 MIC **4k** + 1/64 MIC CFX	0.03
1/16 MIC **4l** + 1/16 MIC AMP	0.14
1/64 MIC **4l** + 1/4 MIC CFX	0.08
1/16 MIC **4m** + 1/16 MIC AMP	0.13
1/32 MIC **4m** + 1/64 MIC CFX	0.05
1/8 MIC **4n** + 1/8 MIC AMP	0.25
1/8 MIC **4n** + 1/64 MIC CFX	0.14
1/16 MIC **4r** + 1/4 MIC AMP	0.31
1/16 MIC **4r** + 1/64 MIC CFX	0.08

**Figure 3:**
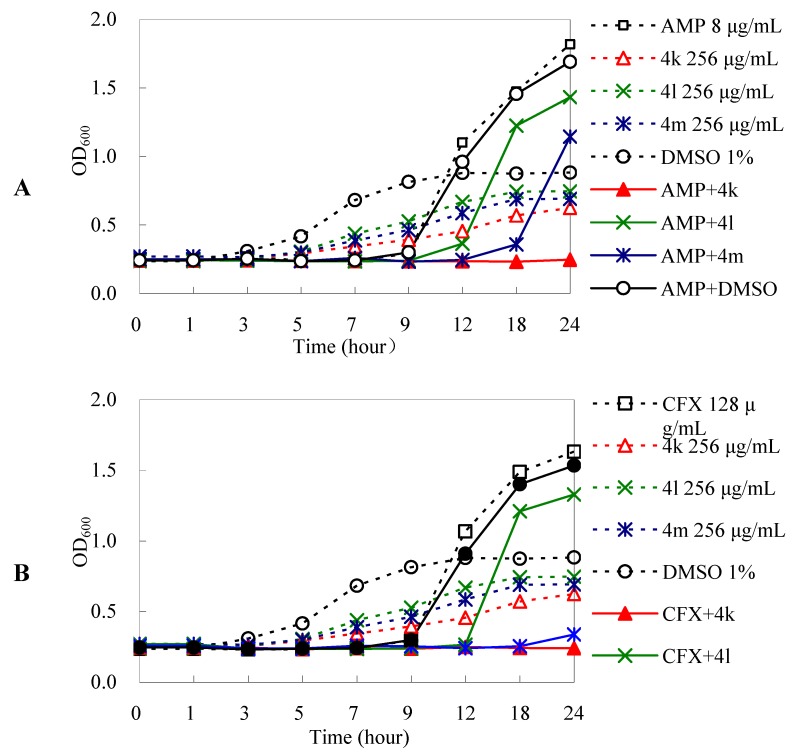
Influence of DHA derivatives on dynamic growth curves of *E. coli* AG100A/pET28a-AcrB. The bacteria from the exponential phase of growth were diluted with LB broth to 1.0 × 10^6^ cfu/mL. Indicated concentrations of DHA derivatives and antibiotics (1/2 MIC) were added into bacterial suspension. Bacterial growth was determined by measuring OD_600_ at regular intervals. AMP, ampicillin; CFX, cefuroxime; **4k**, **4l** and **4m**, DHA derivatives (256 μg/mL, <1/2 MIC) in combinations with AMP (8 μg/mL, 1/2 MIC) or CFX (128 μg/mL, 1/2 MIC). Representative data from one of three independent experiments are shown; the standard deviation bars are not shown.

The results from dynamic bacterial growth assay showed *E. coli* AG100A/pET28a-AcrB grew very well even though antibiotics were added. However, bacterial growth was inhibited by **4k**, **4l** and **4m** in combinations with antibiotics during the exponential phase of growth (from 3 to 9 h) although these three derivatives had no such an effect (Figure 3) alone. Interestingly, the bacterial growth was totally inhibited only by **4k** in combination with ampicillin or cefuroxime from 0 to 12 h; the OD_600_ value was obviously lower than that of antibiotics alone, demonstrating **4k** had the best antibacterial enhancement effect and could be considered as a good EPI candidate.

As well-known, decreased accumulation of antibiotic is the main multidrug resistance mechanism for Gram-negative bacteria. Previously, AS was first found to increase drug accumulation in a dose-dependent manner, suggesting the antibacterial enhancement of AS was tightly associated with increased antibiotic accumulation via targeting of AcrB because AS lost its enhancement activities against AcrB-knocked out *E. coli*. Therefore, AS was thought to be an EPI [12]. Herein, based on the results of molecular docking experiments, twenty-one DHA derivatives were designed and synthesized to imitate with parent nucleus of DHA and succinate tail of AS. Among them, **4k**, **4l**, **4m**, **4n**, and **4r** with new structures exhibited significant synergism with β-lactam antibiotics although they had no directly antibacterial activities themselves. The above results demonstrated these DHA derivatives possessed antibacterial enhancement activities just like AS, suggesting these DHA derivatives were worth further investigation as EPIs.

Considering these DHA derivatives were designed to imitate the parent nucleus of DHA and the succinate tail of AS and they had similar activities to AS, the possible molecular mechanism of these DHA derivatives was thought to also target AcrB to block the AcrAB-TolC efflux pump, leading to increased accumulation of antibiotic. Of course, a series of experiments should be done in the subsequent investigation to prove this.

## 3. Experimental

### 3.1. Materials

#### 3.1.1. Reagents and Apparatus

Precise Micro Melting Point apparatus (Version X-6, Beijing Fukan Instrument Corporation, Ltd, temperature not rectified, Beijing, China); Nuclear Magnetic Resonance spectrometer (Bruker AV-300 MHz, 300 MHz for ^1^H, 75 MHz for ^13^C, Bruker Rheinstetten, Germany); TMS as internal standard); HRMS (Varian 7.0T, Palo Alto, CA, USA). Dihydroartemisinin (analytically pure, Chongqing Holley Wuling Wountain Pharmaceutical Corporation, Ltd, Chongqing, China). Other reagents were all analytically or chemically pure compounds bought from the market and not further processed.

#### 3.1.2. Bacterial Strains

*E. coli* AG100A lacking the gene encoding AcrAB was donated by Professor Hiroshi Nikaido of the University of California (Berkeley, CA, USA). *E. coli* AG100A/pET28a-AcrB re-expressing AcrB, the recombinant AG100A harboring pET28a-AcrB, was constructed in our laboratory.

### 3.2. Methods

#### 3.2.1. Molecular docking

The crystal structure of AcrB [7] (PDB ID, 1IWG) was obtained from the Protein Data Bank, and the three-dimensional structural models of ART, DHA, and AS were drawn in Chem3D Ultra. The molecular docking was performed in MOE 2008 using the Triangle Matcher approach. In the docking calculations of the MOE program, AcrB was defined as the receptor, ART, DHA, and AS were respectively defined as the ligands, other parameters during the docking process were set at default values. Quality assessment of the models was performed using London dG. 

#### 3.2.2. Chemistry

##### 3.2.2.1. Preparation of 10β-(2-Bromoethoxy) Dihydroartemisinin (**3**)

2-Bromoethyl alcohol (23.103 g, 24 mmoL) and Et_2_O (100 mL) were placed into a 250 mL round bottomed flask, and then BF_3_.Et_2_O (4 mL) was added under ice bath cooling. Dihydroartemisinin (15.690 g, 20 mmol) was finally added with stirring. The mixture was stirred for 1.5 h and continuously ice bath cooled. The reaction progress was monitored with TLC. Saturated NaHCO_3_ was added to terminate the completed reaction. The aqueous layer was extracted with EtOAc (30 mL × 2) after liquid separation, and then the organic layers were combined. The organic layer was washed with saturated brine (40 mL), and then dried with anhydrous MgSO_4_, and the solvent was removed through rotary evaporation under reduced pressure. The raw product was recrystallized with a mixed solvent of petroleum ether and EtOAc, and 6.552 g of white crystals were obtained after filtration and vacuum desiccation. The yield was 83.4%; m.p.: 162.0–162.8 °C; 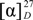 + 138.0 (c 1.0 mg/mL, CH_2_Cl_2_). ^1^H-NMR (CDCl_3_) δ ppm: 0.94 (3H, d, *J* = 6.0 Hz, H-13), 0.96 (3H, d, *J* = 4.8 Hz, H-14), 1.44 (3H, s, H-15), 1.19–2.07 (10H, m, H-2, H-3, H-7, H-8, H-9 and H-10), 2.32–2.42(1H, m, H-1), 2.61–2.71 (1H, m, H-11), 3.52 (2H, t, *J* = 5.7 Hz, H-17), 3.76–3.83 (1H, m, H-16), 4.09–4.17 (1H, m, H-16), 4.85 (1H, d, *J* = 3.6 Hz, H-12), 5.50 (1H, s, H-5); ^13^C-NMR (CDCl_3_) δ ppm: 104.1 (C-4), 102.1 (C-12), 88.1 (C-5), 81.1 (C-6), 68.1 (C-16), 52.6 (C-1), 44.3 (C-7), 37.4 (C-11), 36.4 (C-10), 34.6 (C-3), 31.4 (C-9), 30.9 (C-17), 26.1 (C-8), 24.6 (C-15), 24.3 (C-2), 20.3 (C-14), 13.0 (C-13).

##### 3.2.2.2. Preparation of Dihydroartemisinin Amino Derivatives **4a**–**u**

Compound **3**, CH_3_CN, K_2_CO_3_ and YH were placed in a 100-mL round-bottomed flask, respectively. The mixture reacted at a controlled temperature, while monitoring by TLC. CH_2_Cl_2_ (15 mL) and saturated NaCl solution (20 mL) were added. The aqueous layer was extracted after liquid separation with CH_2_Cl_2_ (10 mL × 2), and then the organic layers were combined, washed with saturated brine (20 mL) and then dried with anhydrous Na_2_SO_4_; CH_2_Cl_2_ was removed through rotary evaporation under reduced pressure. Pure product was obtained after column chromatography. The experimental results are shown in Table 3.

**Table 3:** Experimental results of target compounds **4a**–**u**.

Synthetic Compound	YH	M1/mmol	YH/mmol	K_2_CO_3_/mmol	Temp./°C	Time/h	Product/mmol	Yield/%
**4a**	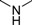	1	6	3	11	32	0.982	98.2
**4b**	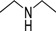	1	6	3	45	7	0.942	94.2
**4c**	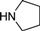	1.5	9	4.5	45	12	1.376	91.7
**4d**	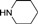	1.5	9	4.5	45	12	1.407	93.8
**4e**	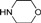	1	6	2	10	16	0.864	86.4
**4f**	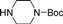	1.5	3	4.5	45	23	1.224	81.6
**4g**	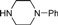	1.5	3	4.5	45	24	1.389	92.6
**4h**	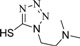	2	3	6	45	6	1.424	71.2
**4i**	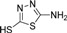	1.5	3	4.5	45	3	1.301	86.7
**4j**	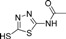	1.5	3	4.5	45	15	1.170	78.0
**4k**	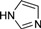	1	4	3	45	34	0.891	89.1
**4l**	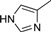	1.5	3	4.5	45	42	1.146	76.4
**4m**	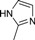	1.5	3	3	45	23	1.146	76.4
**4n**	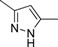	2	3	4	45	6	1.728	86.4
**4o**	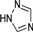	1.5	3	4.5	45	22	1.431	95.4
**4p**	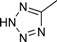	1.5	3	4.5	45	34	0.468	31.2
**4q**	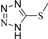	1.5	3	4.5	45	10.5	1.257	83.8
**4r**	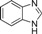	2	3	4	45	12	1.748	87.4
**4s**	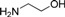	1	6	2	50	13	0.411	41.1
**4t**	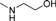	1	6	3	14	24	0.925	92.5
**4u**	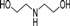	1	6	2	50	21	0.852	85.2

*12β-(2-(Dimethylamino)ethoxy)dihydroartemisinin* (**4a**): Yield: 98.2%; Yellow oil; 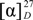 + 139.0 (c 1.1 mg/mL, CHCl_3_). ^1^H-NMR (CDCl_3_) δ ppm: 0.91 (3H, d, *J* = 7.2 Hz, H-14), 0.95 (3H, d, *J* = 6.0 Hz, H-13), 1.44 (3H, s, H-15), 1.22–2.06 (10H, m, H-2, H-3, H-7, H-8, H-9, and H-10), 2.28 (6H, s, H-18), 2.32–2.38 (1H, m, H-1), 2.51–2.54 (2H, m, H-17), 2.60–2.65 (1H, m, H-11), 3.50–3.57 (1H, m, H-16), 3.91–3.98 (1H, m, H-16), 4.80 (1H, d, *J* = 2.7 Hz, H-12), 5.42 (1H, s, H-5).

*12β-(2-(Diethylamino)ethoxy)dihydroartemisinin* (**4b**): Yield: 94.2%; Yellow oil; 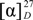 + 116.0 (c 1.1 mg/mL, CHCl_3_). ^1^H-NMR (CDCl_3_) δ ppm: 0.90 (3H, d, *J* = 7.2 Hz, H-14), 0.95 (3H, d, *J* = 6.0 Hz, H-13), 1.05 (6H, t, *J* = 7.5 Hz, H-19), 1.44 (3H, s, H-15), 1.18–2.06 (10H, m, H-2, H-3, H-7, H-8, H-9, and H-10), 2.32–2.42 (1H, m, H-1), 2.57–2.64 (5H, m, H-18 and H-11), 2.69–2.73 (1H, m, H-17), 3.47–3.55 (1H, m, H-16), 3.91–3.99 (1H, m, H-16), 4.79 (1H, s, H-12), 5.42 (1H, s, H-5); ^13^C-NMR (CDCl_3_) δ ppm: 104.0 (C-4), 102.1 (C-12), 87.8 (C-5), 81.1 (C-6), 66.7 (C-16), 52.5 (C-1), 52.1 (C-17), 47.4 (C-18), 44.4 (C-7), 37.4 (C-11), 36.4 (C-10), 34.6 (C-3), 30.8 (C-9), 26.2 (C-8), 24.7 (C-15), 24.3 (C-2), 20.4 (C-14), 13.0 (C-13), 11.8 (C-19).

*12β-(2-Pyrrolidinoethoxy)dihydroartemisinin* (**4c**): Yield: 91.7%; Brown oil; 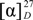 + 140.0 (c 1.1 mg/mL, CH_2_Cl_2_). ^1^H-NMR (CDCl_3_) δ ppm: 0.91 (3H, d, *J* = 7.5 Hz, H-13), 0.95 (3H, d, *J* = 6.3 Hz, H-14), 1.44 (3H, s, H-15), 1.22–2.06 (14H, m, H-2, H-3, H-7, H-8, H-9, H-10, H-18 and H-19), 2.32–2.42 (1H, m, H-1), 2.59–2.80 (7H, m, H-11, H-17 and H-18), 3.54–3.61 (1H, m, H-16), 3.95–4.02 (1H, m, H-16), 4.61 (1H, s, H-12), 5.44 (1H, s, H-5); ^13^C-NMR (CDCl_3_) δ ppm: 104.0 (C-4), 102.0 (C-12), 87.8 (C-5), 81.0 (C-6), 67.1 (C-16), 55.3 (C-17) 54.5 (C-18), 52.5 (C-1), 44.4 (C-7), 37.4 (C-11), 36.4 (C-10), 34.6 (C-3), 30.8 (C-9), 26.1 (C-8), 24.6 (C-15), 24.4 (C-2), 23.4 (C-19), 20.3 (C-14), 13.0 (C-13).

*12β-(2-Piperidinoethoxy)dihydroartemisinin* (**4d**): Yield: 93.8%; m.p.: 70.8–72.1 °C; 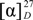 + 135.0 (*c* 1.0 mg/mL, CHCl_3_). ^1^H-NMR (CDCl_3_) δ ppm: 0.90 (3H, d, *J* = 7.2 Hz, H-13), 0.96 (3H, d, *J* = 6.0 Hz, H-14), 1.44 (3H, s, H-15), 1.22–2.06 (16H, m, H-2, H-3, H-7, H-8, H-9, H-10, H-19 and H-20), 2.32–2.60 (8H, m, H-1, H-11, H-17 and H-18), 3.55–3.59 (1H, m, H-16), 3.92–4.00 (1H, m, H-16), 4.79 (1H, s, H-12), 5.44 (1H, s, H-5).

*12β-(2-Morpholinoethoxy)dihydroartemisinin* (**4e**): Yield: 86.4%; Yellow syrup; 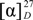 + 59.0 (*c* 1.0 mg/mL, CHCl_3_). ^1^H-NMR (CDCl_3_) δ ppm: 0.90 (3H, d, *J* = 7.5 Hz, H-13), 0.96 (3H, d, *J* = 6.3 Hz, H-14), 1.44 (3H, s, H-15), 1.26–2.05 (10H, m, H-2, H-3, H-7, H-8, H-9 and H-10), 2.32–2.42 (1H, m, H-1), 2.49–2.63 (7H, m, H-11, H-17 and H-18), 3.54–3.62 (1H, m, H-16), 3.72 (3H, t, *J* = 4.5 Hz, H-19), 3.94–4.01(1H, m, H-16), 4.81 (1H, d, *J* = 3.3 Hz, H-12), 5.47 (1H, s, H-5); ^13^C-NMR (CDCl_3_) δ ppm: 104.0 (C-4), 102.0 (C-12), 87.9 (C-5), 81.1 (C-6), 66.9 (C-19), 65.6 (C-16), 58.2 (C-17) 53.8 (C-18), 52.5 (C-1), 44.4 (C-7), 37.5 (C-11), 36.4 (C-10), 34.6 (C-3), 30.8 (C-9), 26.2 (C-8), 24.7 (C-15), 24.4 (C-2), 20.4 (C-14), 13.0 (C-13).

*10β-(2-(4-N-Boc-piperazino)ethoxy))dihydroartemisinin* (**4f**): Yield: 81.6%; m.p.: 107.8–109.1 °C; 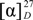 +122.0 (c 1.0 mg/mL, CH_2_Cl_2_). ^1^H-NMR (CDCl_3_) δ ppm: 0.90 (3H, d, *J* = 7.2 Hz, H-13), 0.96 (3H, d, *J* = 6.0 Hz, H-14), 1.43 (3H, s, H-15), 1.46 (9H, s, H-22), 1.22–2.06 (16H, m, H-2, H-3, H-7, H-8, H-9 and H-10), 2.32–2.65 (8H, m, H-1, H-11, H-17 and H-18), 3.40–3.44 (4H, m, H-19), 3.52–3.58 (1H, m, H-16), 3.92–4.00 (1H, m, H-16), 4.80 (1H, s, H-12), 5.46 (1H, s, H-5); ^13^C-NMR (CDCl_3_) δ ppm*:* 154.6 (C-21), 104.0 (C-4), 101.9 (C-12), 87.8 (C-5), 81.0 (C-6), 79.7 (C-22), 66.0 (C-16), 57.9 (C-17), 53.5 (C-19), 52.0 (C-1), 49.2 (C-18), 44.4 (C-7), 37.4 (C-11), 36.4 (C-10), 34.6 (C-3), 30.8 (C-9), 28.4 (C-23), 26.4 (C-17), 26.1 (C-8), 24.6 (C-15), 24.4 (C-2), 20.4 (C-14), 13.0 (C-13).

*12β-(2-(4-N-Phenylpiperazino)* *ethoxy))dihydroartemisinin* (**4g**): Yield: 92.6%; m.p.: 90.0–91.7 °C; 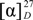 +74.0 (c 1.0 mg/mL, CH_2_Cl_2_). ^1^H-NMR (CDCl_3_) δ ppm: 0.90 (3H, d, *J* = 7.2 Hz, H-13), 0.95 (3H, d, *J* = 6.0 Hz, H-14), 1.44 (3H, s, H-15), 1.23–2.06 (10H, m, H-2, H-3, H-7, H-8, H-9, and H-10), 2.32–2.42 (1H, m, H-1), 2.59–2.70 (7H, m, H-11, H-17 and H-18), 3.21 (4H, t, *J* = 7.5 Hz, H-19), 3.58–3.65 (1H, m, H-16), 3.98–4.05 (1H, m, H-16), 4.83 (1H, d, *J* = 2.7 Hz, H-12), 5.48 (1H, s, H-5), 6.89 (1H, t, *J* = 7.2 Hz, H-23), 6.94 (2H, d, *J* = 7.8 Hz, H-21), 7.25–7.30 (2H, m, H-22); ^13^C-NMR (CDCl_3_) δ ppm*:* 151.3 (C-20), 129.1 (C-22), 120.0 (C-23), 116.0 (C-21), 104.0 (C-4), 102.0 (C-12), 87.9 (C-5), 81.1 (C-6), 66.0 (C-16), 57.9 (C-17), 53.5 (C-19), 52.6 (C-1), 49.2 (C-18), 44.4 (C-7), 37.5 (C-11), 36.4 (C-10), 34.7 (C-3), 30.9 (C-9), 26.2 (C-8), 24.7 (C-15), 24.4 (C-2), 20.3 (C-14), 13.1 (C-13); HRMS calcd for C_27_H_40_N_2_O_5_ (M+H)^+^ 473.3010, found 473.3014.

*12β-(2-((1-(2-(Dimethylamino)ethyl)-1H-tetrazol-5-yl)thio)ethoxy)dihydroartemisinin* (**4h**): Yield: 71.2%; m.p.: 67.9-69.2 °C; 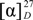 + 72.0 (c 1.0 mg/mL, CHCl_3_). ^1^H-NMR (CDCl_3_) δ ppm: 0.90 (3H, d, *J* = 7.2 Hz, H-13), 0.96 (3H, d, *J* = 5.7 Hz, H-14), 1.44 (3H, s, H-15), 1.20–2.06 (10H, m, H-2, H-3, H-7, H-8, H-9 and H-10), 2.29 (6H, s, H-21), 2.36–2.42(1H, m, H-1), 2.59–2.65(1H, m, H-11), 2.79 (2H, t, *J* = 6.6 Hz, H-20), 3.60 (2H, t, *J* = 5.7 Hz, H-17), 3.74–3.81 (1H, m, H-16), 4.11–4.18 (1H, m, H-16), 4.32 (2H, t, *J* = 6.6 Hz, H-19), 4.82 (1H, s, H-12), 5.42 (1H, s, H-5); ^13^C-NMR (CDCl_3_) δ ppm: 154.1 (C-18), 104.1 (C-4), 102.2 (C-12), 88.0 (C-5), 80.9 (C-6), 66.5 (C-16), 57.2 (C-20), 52.4 (C-1), 45.1 (C-21), 44.2 (C-7), 37.4 (C-11), 36.3 (C-10), 34.5 (C-3), 33.7 (C-17), 30.7 (C-9), 26.1 (C-8), 24.6 (C-15), 24.4 (C-2), 20.3 (C-14), 12.9 (C-13).

*12β-(2-((5-Amino-1,3,4-thiadiazol-2-yl)thio)ethoxy)dihydroartemisinin* (**4i**): Yield: 86.7%; m.p.: 120.1–121.7 °C; 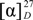 + 59.0 (c 1.0 mg/mL, CHCl_3_). ^1^H-NMR (CDCl_3_) δ ppm: 0.91 (3H, d, *J* = 7.2 Hz, H-13), 0.96 (3H, d, *J* =6.0 Hz, H-14), 1.43 (3H, s, H-15), 1.22–2.06 (10H, m, H-2, H-3, H-7, H-8, H-9 and H-10), 2.32–2.42 (1H, m, H-1), 2.56–2.67 (1H, m, H-11), 3.41 (2H, t, *J* = 5.7 Hz, H-17), 3.70–3.77 (1H, m, H-16), 4.06–4.14 (1H, m, H-16), 4.82 (1H, s, H-12), 5.44 (1H, s, H-5); ^13^C-NMR (DMSO and CDCl_3_) δ ppm: 103.3 (C-4), 101.3 (C-12), 87.2 (C-5), 80.4 (C-6), 66.1 (C-16), 52.0 (C-1), 43.8 (C-7), 36.7 (C-11), 35.9 (C-10), 34.6 (C-3), 34.1 (C-17), 30.3 (C-9), 25.6 (C-8), 24.1 (C-15), 23.8 (C-2), 20.0 (C-14), 12.6 (C-13); HRMS calcd for C_19_H_29_N_3_O_5_S_2_ (M+Na)^+^ 466.1441, found 466.1440.

*12β-(2-((5-Acetamino-1,3,4-thiadiazol-2-yl)thio)ethoxy)dihydroartemisinin* (**4j**): Yield: 78.0%; m.p.: 108.7–110.1 °C; 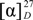 + 83.0 (c 1.0 mg/mL, CHCl_3_). ^1^H-NMR (CDCl_3_) δ ppm: 0.89 (3H, d, *J* = 7.2 Hz, H-13), 0.94 (3H, d, *J* =6.0 Hz, H-14), 1.42 (3H, s, H-15), 1.22–2.05 (10H, m, H-2, H-3, H-7, H-8, H-9 and H-10), 2.32–2.42 (1H, m, H-1), 2.49 (3H, s, H-21), 2.58–2.64 (1H, m, H-11), 3.44–3.57 (2H, m, H-17), 3.75–3.81 (1H, m, H-16), 4.13–4.20 (1H, m, H-16), 4.84 (1H, s, H-12), 5.42 (1H, s, H-5), 13.08 (1H, s, H-20); ^13^C-NMR (CDCl_3_) δ ppm: 168.5 (C-20), 104.1 (C-4), 102.2 (C-12), 88.0 (C-5), 81.0 (C-6), 66.5 (C-16), 52.5 (C-1), 44.3 (C-7), 37.4 (C-11), 36.3 (C-10), 34.6 (C-3), 34.1 (C-17), 30.8 (C-9), 26.1 (C-8), 24.6 (C-15), 24.4 (C-2), 23.0 (C-21), 20.3 (C-14), 12.9 (C-13); HRMS calcd for C_21_H_31_N_3_O_6_S_2_ (M+Na)^+^ 508.1546, found 508.1540.

*12β-(2-(1H-Imidazol-1yl)ethoxy)dihydroartemisinin* (**4k**): Yield: 89.1%; Yellow oil; 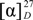 + 68.0 (c 1.1 mg/mL, CHCl_3_). ^1^H-NMR (CDCl_3_) δ ppm: 0.85 (3H, d, *J* =7.2 Hz, H-14), 0.94 (3H, d, *J* = 6.1 Hz, H-13), 1.44 (3H, s, H-15), 1.22–2.07 (10H, m, H-2, H-3, H-7, H-8, H-9, and H-10), 2.32–2.38 (1H, m, H-1), 2.61–2.63 (1H, m, H-11), 3.62–3.68 (1H, m, H-16), 4.15–4.21 (3H, m, H-16 and H-17), 4.76 (1H, s, H-12), 5.11 (1H, s, H-5). 7.00 (1H, s, H-19), 7.09 (1H, s, H-18), 7.62 (1H, s, H-20); ^13^C-NMR (CDCl_3_) δ ppm: 128.7 (C-20), 123.1 (C-19), 118.9 (C-18), 104.1 (C-4), 102.0 (C-12), 87.8 (C-5), 80.8 (C-6), 67.0 (C-16), 52.3 (C-1), 47.2 (C-17), 44.0 (C-7), 37.2 (C-11), 36.3 (C-10), 34.4 (C-3), 30.6 (C-9), 26.1 (C-8), 24.6 (C-15), 24.3 (C-2), 20.3 (C-14), 13.0 (C-13); HRMS calcd for C_20_H_30_N_2_O_5_ (M+H)^+^ 379.2228, found 379.2221.

*12β-(2-(4-Methyl-1H-imidazol-1-yl)ethoxy)dihydroartemisinin* (**4l**): Yield: 76.4%; Yellow oil; 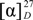 +58.0 (c 1.1 mg/mL, CHCl_3_). ^1^H-NMR (CDCl_3_) δ ppm: 0.91(3H, d, *J* = 6.9 Hz, H-14), 0.95 (3H, d, *J* = 6.3 Hz, H-13), 1.45 (3H, s, H-15), 1.19–2.06 (10H, m, H-2, H-3, H-7, H-8, H-9 and H-10), 2.12 (3H, s, H-21), 2.32–2.43 (1H, m, H-1), 2.59–2.66 (1H, m, H-11), 3.40–3.52 (4H, m, H-16 and H-17), 4.69 (1H, d, *J* = 2.7 Hz, H-12), 5.39 (1H, s, H-5); ^13^C-NMR (CDCl_3_) δ ppm: 127.8 (C-18), 127.2 (C-20), 118.8 (C-19), 104.2 (C-4), 102.0 (C-12), 87.8 (C-5), 80.9 (C-6), 66.9 (C-16), 52.4 (C-1), 47.8 (C-17), 44.0 (C-7), 37.2 (C-11), 36.3 (C-10), 34.4 (C-3), 30.6 (C-9), 26.1 (C-8), 24.6 (C-15), 24.2 (C-2), 20.3 (C-14), 13.0 (C-13), 12.7 (C-21); HRMS calcd for C_21_H_32_N_2_O_5_ (M+H)^+^ 393.2384, found 393.2382.

*12β-(2-(2-Methyl-1H-imidazol-1-yl)ethoxy)dihydroartemisinin* (**4m**): Yield: 76.4%; Yellow oil; 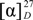 + 52.0 (c 1.1 mg/mL, CHCl_3_). ^1^H-NMR (CDCl_3_) δ ppm: 0.85 (3H, d, *J* =7.2 Hz, H-14), 0.94 (3H, d, *J* = 4.8 Hz, H-13), 1.43 (3H, s, H-15), 1.22–2.07 (10H, m, H-2, H-3, H-7, H-8, H-9, and H-10), 2.29–2.34 (1H, m, H-1), 2.41 (3H, s, H-21), 2.61–2.63(1H, m, H-11), 3.58–3.63 (1H, m, H-16), 3.99-4.16 (3H, m, H-16 and H-17), 4.76 (1H, d, *J* = 3.3 Hz, H-12), 5.08 (1H, s, H-5). 6.88 (1H, s, H-19), 6.92 (1H, s, H-18); ^13^C-NMR (CDCl_3_) δ ppm: 126.7 (C-20 and C-19), 118.9 (C-18), 104.2 (C-4), 102.0 (C-12), 87.8 (C-5), 80.9 (C-6), 66.8 (C-16), 52.4 (C-1), 45.8 (C-17), 44.0 (C-7), 37.2 (C-11), 36.3 (C-10), 34.4 (C-3), 30.6 (C-9), 26.1 (C-8), 24.6 (C-15), 24.2 (C-2), 20.3 (C-14), 13.0 (C-13), 12.9 (C-21); HRMS calcd for C_21_H_32_N_2_O_5_ (M+H)^+^ 393.2384, found 393.2386.

*12β-(2-(3,5-Dimethyl-1H-pyrazol-1-yl)ethoxy)dihydroartemisinin* (**4n**): Yield: 86.4%; Yellow oil; 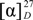 + 49.0 (c 1.0 mg/mL, CH_2_Cl_2_). ^1^H-NMR (CDCl_3_) δ ppm: 0.81 (3H, d, *J* = 7.2 Hz, H-14), 0.87 (3H, d, *J* = 6.3Hz, H-13), 1.42 (3H, s, H-15), 1.26–2.08 (10H, m, H-2, H-3, H-7, H-8, H-9, and H-10), 2.29–2.37 (1H, m, H-1), 2.21 (3H, s, H-21), 2.28 (3H, s, H-22), 2.51–2.59 (1H, m, H-11), 3.64–3.69 (1H, m, H-16), 4.12–4.28 (3H, m, H-16 and H-17), 4.75 (1H, d, *J* = 2.7 Hz, H-12), 5.07 (1H, s, H-5), 5.86 (1H, s, H-19); ^13^C-NMR (CDCl_3_) δ ppm: 147.4 (C-20), 139.4 (C-20), 104.6 (C-4), 103.9 (C-19), 101.7 (C-12), 87.6 (C-5), 80.9 (C-6), 66.4 (C-16), 52.3 (C-1), 47.9 (C-17), 44.2 (C-7), 37.0 (C-11), 36.3 (C-10), 34.6 (C-3), 30.7 (C-9), 26.1 (C-8), 24.5 (C-15), 24.0 (C-2), 20.2 (C-14), 13.0 (C-13), 12.8 (C-21), 11.1 (C-22); HRMS calcd for C_22_H_34_N_2_O_5_ (M+Na)^+^ 429.2360, found 429.2356.

*12β-(2-(1H-1,2,4-Triazol-1-yl)ethoxy)dihydroartemisinin* (**4o**): Yield: 95.4%; m.p.: 93.5–95.2 °C; 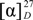 + 96.0 (c 1.1 mg/mL, CHCl_3_). ^1^H-NMR (CDCl_3_) δ ppm: 0.78 (3H, d, *J* =7.2 Hz, H-14), 0.94 (3H, d, *J* = 4.5 Hz, H-13), 1.42 (3H, s, H-15), 1.19–2.08 (10H, m, H-2, H-3, H-7, H-8, H-9, and H-10), 2.31–2.39 (1H, m, H-1), 2.58–2.62 (1H, m, H-11), 3.74–3.81 (1H, m, H-16), 4.09–4.17 (1H, m, H-16), 4.36–4.38 (2H, m, H-17), 4.78 (1H, s, H-12), 5.17 (1H, s, H-5), 7.96 (1H, s, H-19), 8.12 (1H, s, H-18).

*12β-(2-((5-Methyl-2H-tetrazol-2-yl)ethoxy)dihydroartemisinin* (**4p**): Yield: 31.2%; m.p.:114.2–115.7 °C; 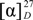 + 93.0 (c 1.1 mg/mL, CHCl_3_). ^1^H-NMR (CDCl_3_) δ ppm: 0.75 (3H, d, *J* = 7.2 Hz, H-14), 0.94 (3H, d, *J* = 4.5 Hz, H-13), 1.42 (3H, s, H-15), 1.21–2.04 (10H, m, H-2, H-3, H-7, H-8, H-9, and H-10), 2.29–2.38 (H, m, H-1), 2.57 (3H, s, H-19), 2.58–2.61 (H, m, H-11), 3.80–3.87 (1H, m, H-16), 4.34–4.37 (1H, m, H-16), 4.46–4.48 (2H, m, H-17), 4.76 (1H, s, H-12), 5.11 (1H, s, H-5); ^13^C-NMR (CDCl_3_) δ ppm: 152.1 (C-18), 104.2 (C-4), 102.3 (C-12), 87.7 (C-5), 80.7 (C-6), 65.8 (C-16), 52.3 (C-1), 47.0 (C-17), 43.9 (C-7), 37.2 (C-11), 36.2 (C-10), 34.3 (C-3), 30.5 (C-9), 26.0 (C-8), 24.5 (C-15), 24.2 (C-2), 20.3 (C-14), 13.0 (C-13), 8.9 (C-19); HRMS calcd for C_19_H_30_N_4_O_5_ (M+Na)^+^ 417.2108, found 417.2106.

*12β-(2-(5-(Methylthio)-2H-tetrazol-2-yl)ethoxy)dihydroartemisinin* (**4q**): Yield: 83.8%; m.p.: 121.7–123.3 °C; 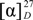 + 126.0 (c 1.0 mg/mL, CHCl_3_). ^1^H-NMR (CDCl_3_) δ ppm: 0.90 (3H, d, *J* =7.5 Hz, H-14), 0.96 (3H, d, *J* = 6.3 Hz, H-13), 1.43 (3H, s, H-15), 1.21–2.04 (10H, m, H-2, H-3, H-7, H-8, H-9 and H-10), 2.32–2.43 (H, m, H-1), 2.61–2.66 (H, m, H-11), 3.63 (2H, t, *J* =5.7 Hz, H-17), 3.75–3.83 (1H, m, H-16), 3.93 (3H, s, H-19), 4.12–4.19 (H, m, H-16), 4.83 (1H, d, *J* =2.7 Hz, H-12), 5.43 (1H, s, H-5); ^13^C-NMR (CDCl_3_) δ ppm: 154.0 (C-18), 104.1 (C-4), 102.2 (C-12), 87.9 (C-5), 80.9 (C-6), 66.5 (C-16), 52.4 (C-1), 44.2 (C-7), 37.4 (C-11), 36.3 (C-10), 34.5 (C-3), 33.6 (C-17), 33.4 (C-19), 30.7 (C-9), 26.1 (C-8), 24.6 (C-15), 24.3 (C-2), 20.3 (C-14), 12.9 (C-13); HRMS calcd for C_19_H_30_N_4_O_5_S (M+Na)^+^ 449.1829, found 449.1821.

*12β-(2-(1H-Benzoimidazol-1-yl)ethoxy)dihydroartemisinin* (**4r**): Yield: 87.4%; Yellow oil; 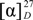 + 94.0 (c 1.0 mg/mL, CH_2_Cl_2_). ^1^H-NMR (CDCl_3_) δ ppm: 0.76 (3H, d, *J* = 7.5 Hz, H-14), 0.83 (3H, d, *J* = 6.0 Hz, H-13), 1.39 (3H, s, H-15), 1.15–1.99 (10H, m, H-2, H-3, H-7, H-8, H-9 and H-10), 2.24–2.35 (H, m, H-1), 2.52–2.59 (H, m, H-11), 3.70–3.76 (1H, m, H-16), 4.28–4.43 (3H, m, H-16 and H-17), 4.73 (1H, d, *J* =2.7 Hz, H-12), 4.91 (1H, s, H-5), 7.30 (2H, t, *J* =2.4 Hz, H-21 and H-22), 7.42 (1H, d, *J* =7.5 Hz, H-23), 7.80 (1H, d, *J* =7.5 Hz, H-20), 7.95 (1H, s, H-18); ^13^C-NMR (CDCl_3_) δ ppm: 143.5 (C-18), 143.3 (C-19), 133.6 (C-24), 122.9 (C-20), 122.1 (C-21), 120.2 (C-22), 109.6 (C-23), 104.0 (C-4), 102.0 (C-12), 87.6 (C-5), 80.7 (C-6), 65.7 (C-16), 52.2 (C-1), 44.8 (C-17), 43.9 (C-7), 37.0 (C-11), 36.2 (C-10), 34.2 (C-3), 30.5 (C-9), 26.0 (C-8), 24.4 (C-15), 24.2 (C-2), 20.1 (C-14), 12.8 (C-13).

*12β-(2-((2-Hydroxyethyl)amino)ethoxy)* *dihydroartemisinin* (**4s**): Yield: 44.1%; Yellow oil; 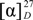 + 87.0 (c 1.0 mg/mL, CHCl_3_). ^1^H-NMR (CDCl_3_) δ ppm: 0.91(3H, d, *J* = 7.2 Hz, H-14), 0.95 (3H, d, *J* = 6.0 Hz, H-13), 1.44 (3H, s, H-15), 1.22–2.06 (10H, m, H-2, H-3, H-7, H-8, H-9 and H-10), 2.38–2.47(1H, m, H-1), 2.58–2.73 (5H, m, H-11, H-17 and H-18), 3.49–3.59(3H, m, H-16 and H-19), 3.90–3.98 (1H, m, H-16), 4.80 (1H, s, H-12), 5.42 (1H, s, H-5).

*12β-(2-((2-Hydroxyethyl)(methyl)amino)ethoxy)dihydroartemisinin* (**4t**): Yield: 92.5%; Yellow oil; 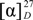 + 182.0 (*c* 1.0 mg/mL, CHCl_3_). ^1^H-NMR (CDCl_3_) δ ppm: 0.91 (3H, d, *J* = 7.2 Hz, H-14), 0.95 (3H, d, *J* = 6.0 Hz, H-13), 1.44 (3H, s, H-15), 1.22–2.06 (10H, m, H-2, H-3, H-7, H-8, H-9 and H-10), 2.32 (3H, s, H-21), 2.37–2.47 (1H, m, H-1), 2.58–2.73 (5H, m, H-11, H-17 and H-18), 3.49–3.59 (3H, m, H-16 and H-19), 3.90–3.98 (1H, m, H-16), 4.80 (1H, s, H-12), 5.42 (1H, s, H-5); ^13^C-NMR (CDCl_3_) δ ppm: 104.1(C-4), 102.2 (C-12), 87.9 (C-5), 81.0 (C-6), 66.1 (C-16), 58.9 (C-19), 58.2 (C-17), 56.9 (C-18), 52.5 (C-1), 44.3 (C-7), 42.0 (C-21), 37.4 (C-11), 36.4 (C-10), 34.6 (C-3), 30.8 (C-9), 26.1 (C-8), 24.6 (C-15), 24.4 (C-2), 20.4 (C-14), 13.0 (C-13); HRMS calcd for C_20_H_35_NO_6_ (M+H)^+^ 386.2537, found 386.2545.

*12β-(2-(Bis(2-hydroxyethyl)amino)ethoxy)dihydroartemisinin* (**4u**): Yield: 85.2%; Yellow syrup; 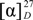 + 88.0 (*c* 1.0 mg/mL, CHCl_3_). ^1^H-NMR (CDCl_3_) δ ppm: 0.91 (3H, d, *J* = 7.2 Hz, H-14), 0.95 (3H, d, *J* = 6.0 Hz, H-13), 1.44 (3H, s, H-15), 1.27–2.06 (10H, m, H-2, H-3, H-7, H-8, H-9 and H-10), 2.32–2.42 (1H, m, H-11), 2.65–2.69 (1H, m, H-1), 2.89–2.97 (6H, m, H-17 and H-18), 3.58–3.62 (1H, m, H-16), 3.67–3.73 (4H, m, H-19), 3.99–4.03 (1H, m, H-16), 4.83 (1H, s, H-12), 5.44 (1H, s, H-5); ^13^C-NMR (CDCl_3_) δ ppm: 104.2 (C-4), 102.4 (C-12), 87.9 (C-5), 80.9 (C-6), 65.6 (C-16), 58.8 (C-19), 54.3 (C-17), 56.4 (C-18), 52.5 (C-1), 44.2 (C-7), 37.3 (C-11), 36.3 (C-10), 34.5 (C-3), 30.7 (C-9), 26.1 (C-8), 24.6 (C-15), 24.4 (C-2), 20.3 (C-14), 13.0 (C-13); HRMS calcd for C_21_H_37_NO_7_ (M+Na)^+^ 438.2462, found 438.2456.

#### 3.2.3. Antibacterial Enhancement Effect

##### 3.2.3.1. Bacterial Growth

Single colony from Luria-Bertani (LB) agar plates was transferred to sterile liquid LB broth (10 g/L tryptone, 10 g/L NaCl and 5 g/L yeast extract) and cultivated aerobically in a 50 mL volume at 37 °C in a heated, shaking and environmental chamber for 12 h. These cultures were then transferred to 500 mL of fresh LB broth for another 12 h. When bacteria were in the exponential phase of growth, the suspension was centrifuged at 1500 g for 5 min at 37 °C and the supernatant was discarded. The bacteria were re-suspended and diluted in fresh LB broth to achieve a concentration of ~1.0 × 10^10^ cfu/mL.

##### 3.2.3.2. Drug Susceptibility Assay

Bacteria in the exponential phase of growth (1 × 10^5^ cfu/mL) were inoculated into 96-well plates. MICs were determined by serial 2-fold dilutions in LB broth containing different drugs in accordance with the CLSI (formerly NCCLS, 2010). The MICs of combined drugs were also assayed. Synergy, or otherwise, was determined using the fractional inhibitory concentration index (FICI).

##### 3.2.3.3. Dynamic Bacterial Growth Assay

Bacteria in the exponential phase of growth were diluted in LB broth to reach a concentration of 1.0 × 10^6^ cfu/mL. According to the MIC results, different concentrations of DHA derivatives and antibiotics were added into bacterial suspensions. The bacterial growth was determined by measuring OD_600_ at regular intervals.

## 4. Conclusions

In conclusion, molecular docking experiments showed that ART, AS and DHA could dock into AcrB very well, especially DHA and AS; both DHA and AS had same docking pose. The affinity between AS and AcrB seemed weaker than that of DHA, while the succinate tail of AS, like a “bug”, could extend in the binding pocket very well. Imitating the parent nucleus of DHA and the succinate tail of AS, twenty-one DHA derivatives **4a**–**u** were designed, synthesized and evaluated. The synergistic activities against *E. coli* AG100A/pET28a-AcrB showed that the novel compounds **4k**, **4l**, **4m**, **4n**, and **4r** possessed significant synergism in combination with β-lactam antibiotics although they themselves had no direct antibacterial activity. Among these five DHA derivatives, **4k** had the best antibacterial enhancement effect. In conclusion, our results provided a new idea and several candidate antibacterial enhancer compounds against multidrug resistant *E. coli*.
